# Copy number alteration profiling facilitates differential diagnosis between ossifying fibroma and fibrous dysplasia of the jaws

**DOI:** 10.1038/s41368-021-00127-3

**Published:** 2021-06-30

**Authors:** Ming Ma, Lu Liu, Ruirui Shi, Jianyun Zhang, Xiaotian Li, Xuefen Li, Jiaying Bai, Jianbin Wang, Yanyi Huang, Tiejun Li

**Affiliations:** 1 Department of Oral Pathology, Peking University School and Hospital of Stomatology & National Center of Stomatology & National Clinical Research Center for Oral Diseases & National Engineering Laboratory for Digital and Material Technology of Stomatology & Beijing Key Laboratory of Digital Stomatology & Research Center of Engineering and Technology for Computerized Dentistry Ministry of Health & NMPA Key Laboratory for Dental Materials, Beijing, China; 2 Research Unit of Precision Pathologic Diagnosis in Tumors of the Oral and Maxillofacial Regions, Chinese Academy of Medical Sciences (2019RU034), Beijing, China; 3 Beijing Advanced Innovation Center for Genomics (ICG), Biomedical Pioneering Innovation Center (BIOPIC), School of Life Sciences, Peking University, Beijing, China; 4 Central Laboratory, Peking University School and Hospital of Stomatology, Beijing, China; 5 School of Life Sciences and Beijing Advanced Innovation Center for Structural Biology, Tsinghua University, Beijing, China; 6 College of Chemistry and Molecular Engineering and Beijing National Laboratory for Molecular Sciences, Peking University, Beijing, China; 7 Institute for Cell Analysis, Shenzhen Bay Laboratory, Guangdong, China

**Keywords:** Pathogenesis, Biological techniques

## Abstract

Ossifying fibroma (OF) and fibrous dysplasia (FD) are two fibro-osseous lesions with overlapping clinicopathological features, making diagnosis challenging. In this study, we applied a whole-genome shallow sequencing approach to facilitate differential diagnosis via precise profiling of copy number alterations (CNAs) using minute amounts of DNA extracted from morphologically correlated microdissected tissue samples. Freshly frozen tissue specimens from OF (*n* = 29) and FD (*n* = 28) patients were obtained for analysis. Lesion fibrous tissues and surrounding normal tissues were obtained by laser capture microdissection (LCM), with ~30–50 cells (5 000–10 000 µm^2^) per sample. We found that the rate of recurrent CNAs in OF cases was much higher (44.8%, 13 of 29) than that in FD cases (3.6%, 1 of 28). Sixty-nine percent (9 of 13) of the CNA-containing OF cases involved segmental amplifications and deletions on Chrs 7 and 12. We also identified eight CNA-associated genes (HILPDA, CALD1, C1GALT1, MICALL2, PHF14, AIMP2, MDM2, and CDK4) with amplified expression, which was consistent with the copy number changes. We further confirmed a jaw lesion with a previous uncertain diagnosis due to its ambiguous morphological features and the absence of GNAS mutation as OF based on the typical Chr 12 amplification pattern in its CNA profile. Moreover, analysis of a set of longitudinal samples collected from an individual with a cellular lesion in suspicion of OF at the first surgery, recurrence and the latest malignant transformation revealed identical CNA patterns at the three time points, suggesting that copy number profiling can be used as an important tool to identify borderline lesions or lesions with malignant potential. Overall, CNA profiling of fibro-osseous lesions can greatly improve differential diagnosis between OF and FD and help predict disease progression.

## Introduction

Benign fibro-osseous lesions, comprising a group of diseases with different etiologies, courses of treatment, and prognoses, are characterized by the replacement of bone by a connective tissue matrix containing varying degrees of mineralization.^1^ According to the latest (4th) edition of the World Health Organization Classification of Head and Neck Tumors (WHO, 2017), fibro-osseous lesions include fibrous dysplasia (FD), ossifying fibroma (OF), familial gigantiform cementoma, and cemento-osseous dysplasia.^2,3^ Among them, the most common lesions are FD and OF of the jaws.^4,5^ FD is a disorder that can affect almost all bones in the body. FD onset commonly occurs during childhood, and its growth is self-limiting, with most lesions tending to exhibit slowed proliferation and becoming static once skeletal development is complete.^6^ Thus, trimming surgery, rather than the complete removal of all lesions, when skeletal maturity is reached has been suggested for most cases of FD.^7,8^ In comparison, OF is an uncommon benign neoplasm that occurs almost anywhere in the facial skeleton, principally in the jaws, and is thought to originate from the periodontal ligament.^9,10^ OF commonly occurs during the third and fourth decades of life,^11,12^ with a disease course varying from slow-growing to aggressive. Under some circumstances, OF may be destructive, causing invisible craniofacial deformity and complications, and completely excising the tumor at the earliest stage is favored by the majority of experts.^13,14^ Although surgery is the mainstay method of treating both FD and OF, treatment of each should be specific given the essential differences between them, making a differential diagnosis of OF and FD crucial.

In general, the final diagnosis of jaw OF or FD, especially regarding the differentiation between them, has been based on an integrative assessment of the clinical manifestations and histopathologic and radiographic features.^15^ Nevertheless, these features frequently overlap, causing uncertain distinctions between the diseases. The identification of low-grade osteosarcomas from OF and FD has become challenging for the same reason.^12,16^ However, it has been reported that activating mutations in GNAS are highly associated with FD but are absent in OF, providing a molecular tool for their differential diagnosis.^5,17^ As GNAS mutations can only be detected in 45%–88% of FD cases,^18,19^ additional methods to better differentiate FD from OF have become vital.

Copy number alteration (CNA) is the major structural alteration ascribed to genomic rearrangement, comprising gains (amplifications) and losses (deletions) that affect the integer copy numbers of various locations of the genome.^20–22^ Many CNA events have been implicated in causing multiple diseases, including various cancers, neuronal degenerate diseases, and metabolic diseases.^23–27^ Recent studies have also shown that CNAs can be present in morphologically normal tissues, indicating the possibility of their serving as a molecular pathological index for diagnosis.

Here, we present a new strategy, namely, multiregional microdissection sequencing (MMS), to combine morphological information with CNA profiles to reduce the ambiguity of differentiation between FD and OF. We applied laser capture microdissection (LCM) to precisely acquire morphologically uniform microscale tissue samples with a cell number of ~30–50. We utilized the Tn5 transposase to construct whole-genome sequencing libraries through direct tagmentation and then generated genome-wide CNA profiles with shallow sequencing (0.1×). Unlike most previous research on tumor copy number profiling, which often measured bulk samples containing mixed cell types, our work accurately depicts copy number changes with high resolution and high precision due to the high cellular purity through morphologically aided LCM. Using MMS, we examined genome CNA profiles in 28 FD patients and 29 OF patients and found that nearly half of the OF cases contained significant copy number changes but that copy numbers were normal in most FD tissues. We also identified Chr 7 and Chr 12 as hotspots for OF-associated copy number changes. Such characteristic CNA patterns, especially the high rates of amplification and deletion on Chr 7 and Chr 12, adds information of genomic integrity to morphological assessments. Our approach is a potentially competent solution for the differential diagnosis of OF and FD and the prediction of disease progression.

## Results

### Clinicopathological findings

**Fig. 1:**
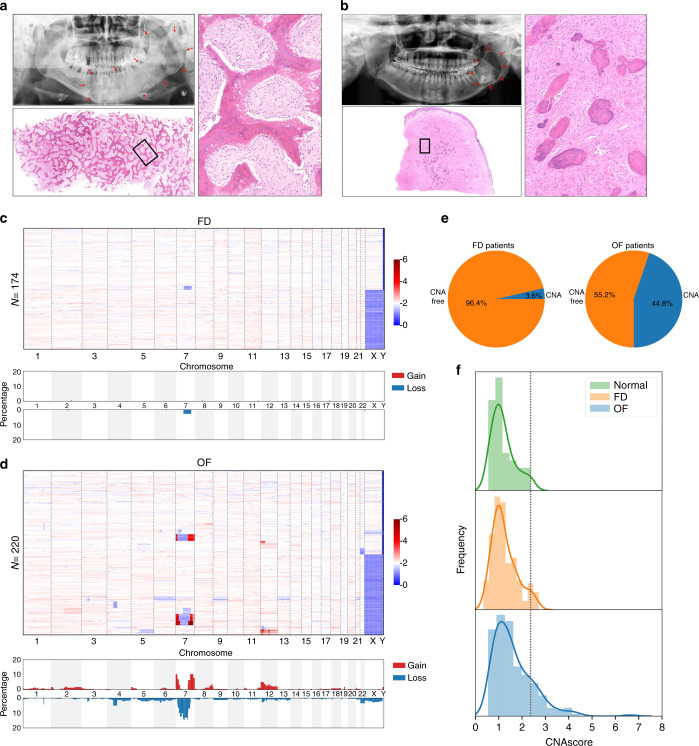
CNA profiling of FD and OF. **a** Panoramic radiograph of fibrous dysplasia revealing a typical ground-glass opacity of the left mandible, with indistinct borders that blend imperceptibly with the surrounding uninvolved bone, indicated by the red arrows. Histologically, fibrous dysplasia is characterized by irregular trabeculae of woven bone that are uniformly distributed throughout the lesion and present various shapes (“O”, “C”, and “V”), without rows of osteoblastic rimming. The image on right shows the rectangular area (bottom left) at higher magnification. Magnification: ×12.5, ×200. **b** Panoramic radiograph of the left posterior mandibular with ossifying fibroma showing a mixed radiolucent and radiopaque lesion with a well-demarcated border, indicated by the red arrows. Histologically, ossifying fibroma presented a variety of calcified structures that featured basophilic circular spherules of osteoids or bone resembling the cementicle. The image on right shows the rectangular area (bottom left) at higher magnification. Magnification: ×12.5, ×200. **c** CNA profiles of 174 samples from 28 FD patients. Heatmap (upper panel) and aggregation of copy number alterations (lower panel) show the key CNA events on specific chromosomes. For the lower panel, the *y* axis represents the percentage of samples harboring CNAs. **d** CNA profiles of 220 samples from 29 OF patients. Heatmap (upper panel) and aggregation of copy number alterations (lower panel) show the key CNA events on specific chromosomes. For the lower panel, the *y* axis represents the percentage of samples harboring CNAs. **e** Ratio of patients with or without CNA in FD (left panel) and OF (right panel). **f** Normalized frequency distribution of CNAscore in normal, FD, and OF samples.

The clinical features of the cases selected in our study are summarized in Supplementary Table 1 and Tables 2, 3. Of the 29 OF cases, 9 were located at the maxilla and 20 at the mandible; the male-to-female ratio was 0.93 (male 14; female 15). The mean age of disease onset was (24.2 ± 15.1) years (range 5 to 61 years), and the average age at surgery was (25.5 ± 15.2) years (range 5–62 years). The disease duration ranged from 0.1 to 7 years, with a mean of (1.4 ± 1.9) years. Of the 28 FD cases, 14 occurred at the maxilla and 5 at the mandible; 7 cases presented both mandibular and maxillary lesions, and 2 patients showed multiple bone lesions involving the jaw and zygoma. Three FD patients were diagnosed with McCune–Albright syndrome. The male-to-female ratio was 1.33 (male 16; female 12). The mean age at FD onset was (16.8 ± 11.0) years (range 2–40 years), and the average age at surgery was (25.4 ± 9.9) years (range 6–48 years). The disease duration ranged from 0.1 to 30 years, with a mean of (8.7 ± 7.3) years. Histologically, FD and OF have distinct morphological features. FD was characterized by loose cellular connective tissue replacing normal bone marrow, and we observed that woven bones were uniformly distributed throughout the lesion and presented various shapes but without osteoblastic rimming (Fig. 1a). OF presented a variety of calcified structures that featured basophilic circular spherules of osteoid or bone resembling the cementicle (Fig. 1b).

### Spatially correlated copy number alteration profiling

**Fig. 2:**
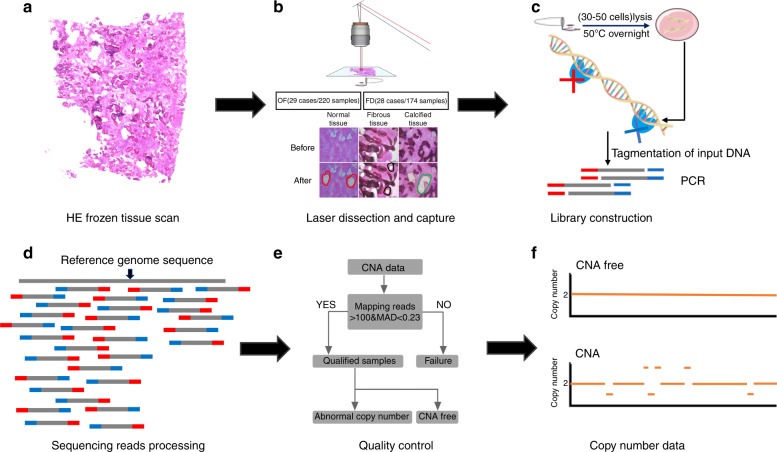
Overview of the multiregional microdissection sequencing (MMS) approach. **a** Frozen tissue sections (10 μm-thick) were stained with hematoxylin and eosin (H&E). **b** Each sample contained 30–50 cells dissected by LCM from 10-µm sections to lysis buffer. **c** Tn5 transposase-assisted sequencing library construction. Released DNA molecules were tagmented, barcoded, and PCR amplified. **d** NGS reads were mapped to the reference genome and counted to infer the copy number ratio. **e** Samples with low quality were filtered out based on amplification noise and mapped reads. **f** Samples with or without CNAs were identified and used to perform downstream analysis.

To obtain CNA profiles of minibulk samples, we applied the MMS strategy, which combines LCM and a low-input whole-genome sequencing library construction process. Briefly, two adjacent sections for each frozen tissue were sliced and stained with H&E. One of the sections was 5 µm and used for morphological identification; the other was 10 µm for LCM (Fig. 2a). Each LCM sample, containing 30–50 morphologically consistent cells, was lysed individually (Fig. 2b). Genomic DNA was tagmented by Tn5 followed by PCR barcoding (Fig. 2c) to index each sample. In each sequencing run, 200–400 barcoded libraries were pooled and sequenced; in this study, we sequenced 471 samples collected from 61 patients.

The average sequencing depth for each sample was 0.3 G (0.1×). To infer copy number profiles, reads were mapped to the reference genome, and the normalized copy number ratio was counted (Fig. 2d). The circular binary segmentation (CBS) algorithm was used (alpha = 0.1, min. width = 5, undo. SD = 0.1) to identify the copy number of each segment (1 Mb). To filter out samples with low quality, the number of mapping reads and median absolute deviation of pairwise difference (MAPD) were applied to evaluate the data quality. Only samples with more than 100 000 mapping reads and MAPD < 0.23 were considered qualified and used for downstream analysis (Fig. 2e, f).

### Copy Number Alteration Profiles of FD and OF

To assess the presence of signature differences in terms of CNAs between FD and OF, we constructed and sequenced 645 minibulk libraries. Among the libraries, 179 samples (FD = 174, normal tissue = 5) from 28 FD patients and 258 (OF = 220, normal tissue = 38) samples from 29 OF patients passed the quality control step. Except for one patient whose samples harbored one copy number loss event on Chr 7, most FD patients (96.4%) were CNA-free (Fig. 1c, e). However, we did find that 13 (44.8%) OF patients harbored CNAs (Fig. 1d, e). These CNAs were distributed across the entire genome, except for Chr 11, 15, 16, 17, and 19. Although CNA patterns showed clear intrapatient heterogeneity among OF samples, we observed that 3 patients shared similar copy number changes on Chr 7 and that 3 patients shared similar patterns on Chr 12. Both hotspots were detected in male and female patients (Fig. 1d).

We applied CNAscore to quantitatively estimate the degree of genome complexity. CNAscore is a combination of two components reflecting deviation of the neutral ploidy value and the degree of chromosomal instability (see “Materials and methods” section). We calculated the CNAscore for 43 normal samples, revealing a narrow distribution ranging from 0.554 to 2.373, with a median of 1.107 (Fig. 1f). The CNAscore for 174 FD samples showed a distribution high similar to that of the normal samples, spanning a range from 0.350 to 2.721, with a median value of 1.108; these results are in accordance with previous observations that most of the FD samples were CNA-free (Fig. 1f). However, the score of 220 OF samples ranged from 0.546 to 6.661 (median 1.420), with 38 samples (17%) having a CNAscore larger than 2.373, further demonstrating the higher degree of genome complexity for OF samples (Fig. 1f).

### Hotspot CNAs in OF patients

**Fig. 3:**
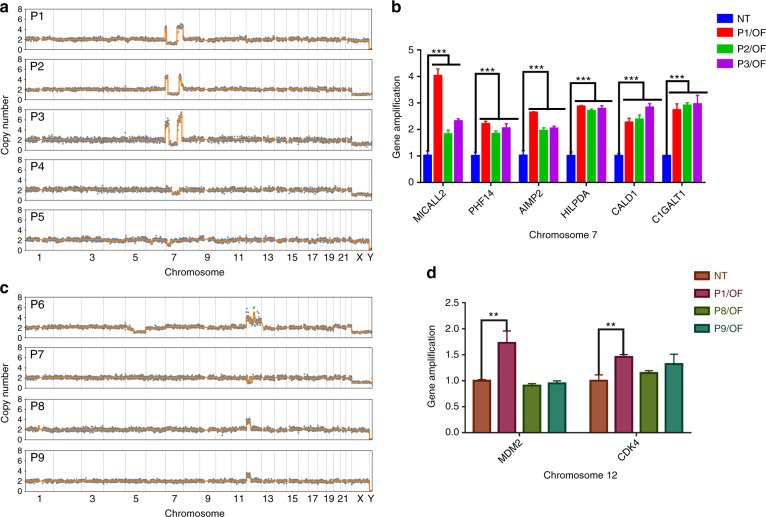
CNA hotspots in OF patients. **a** CNA patterns on Chr 7 of P1, P2, P3, P4, and P5. **b** Results of qPCR showing amplification of the MICALL2, PHF14, AIMP2, HILPDA, CALD1, and C1GALT1 genes, which are located in the CNA area of Chr 7 in P1, P2, and P3. **c** CNA patterns on Chr 12 of P6, P7, P8, and P9. **d** Results of qPCR showing amplification of MDM2 and CDK4, which are located in the CNA area of Chr 12 in P6.

Among OF patients whose lesions contained abnormal copy numbers, 5 (17.2%) harbored CNAs on Chr 7, while 4 (13.8%) harbored CNAs on Chr 12 (Figs. 1d and 3a, c). Similar breakpoint patterns on Chr 7 were found in patients P1, P2, and P3 (Fig. 3a). It is worth noting that the only FD patient (P11) with CNAs showed copy number loss on Chr 7, as OF patients did (Fig. 1c). On Chr 12, copy number changes tended to occur on the short arm, with gains being more prevalent than losses (3-fold).

We then examined whether the recurrence patterns on Chr 7 and Chr 12 were associated with the progression of OF. To identify potential marker genes, we screened genes reported to be associated with cancer progression and located in the common CNA regions on Chr 7 and Chr 12 (Supplementary Tables 4 and 5). Specifically, we identified genetic alterations of HILPDA, CALD1, C1GALT1, MICALL2, PHF14, and AIMP2 on Chr 7, along with MDM2 and CDK4 on Chr 12, by qPCR. These genes are closely associated with the occurrence, development, and therapy of head and neck tumors as well as other tumors. In all three patients with similar breakpoint patterns on Chr 7, significantly higher amplification of these genes than in normal tissues was detected (Fig. 3b). One patient with copy number gains encompassing MDM2 and CDK4 showed amplification in qPCR as well (Fig. 3d). Overall, the qPCR results were consistent with the results for copy number changes.

### Consistency of CNA patterns in fibrous and calcified tissues in OF

**Fig. 4:**
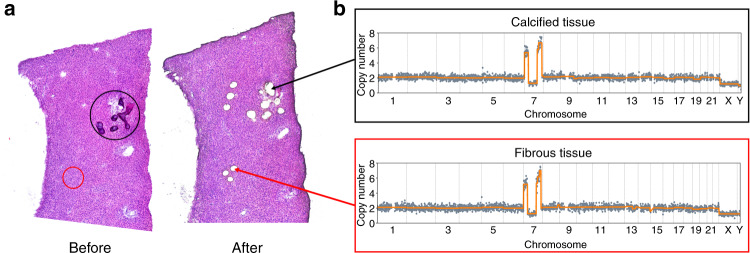
Identification of CNAs in both calcified and fibrous tissues of the same OF section. **a** Whole-tissue scanning of one H&E-stained section (left panel) from OF patient P3 and laser microdissection of fibrous and calcified tissues (right panel). **b** An identical CNA pattern on Chr 7 was identified in both fibrous and calcified tissues in P3.

Using LCM to separate cells with clearly defined morphology and reducing the sufficient cell number to less than 50, we were able to infer clone-specific CNA patterns with spatial information. We clearly identified samples with different patterns in one H&E section, indicating intrapatient heterogeneity. We dissected samples from fibrous and calcified tissues, and the same CNA patterns were found in both 7 OF patients and 1 FD patient (Fig. 4), which may indicate that fibrous and calcified components that share the same CNA patterns are derived from the same clone. It is also possible that both types of cells derived from the same progenitor cell.

### Case study one: differential diagnosis assisted by CNA profiling

The fact that CNAs in OF patients occurred at a significantly higher rate than those in FD patients suggested that CNAs might contribute to differential diagnosis between OF and FD. To test this hypothesis, we studied one case that had been difficult to diagnose.

**Fig. 5:**
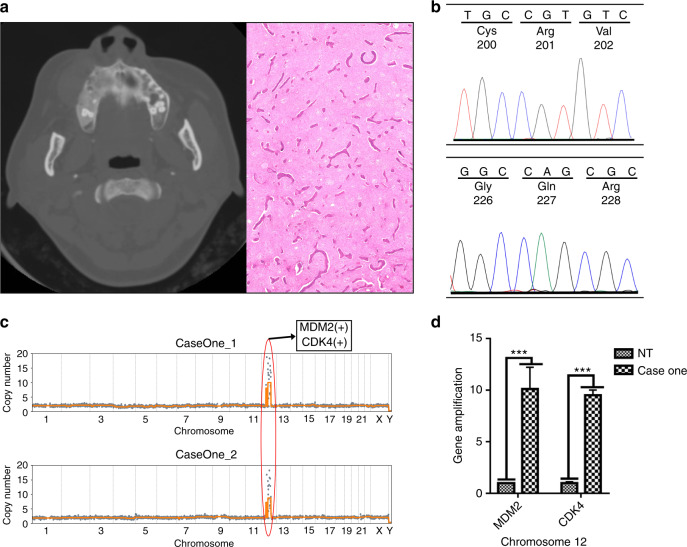
Differential diagnosis for Case 1. **a** An axial view of a CT scan demonstrating a ground-glass opacity on the right maxilla with buccal expansion involving upper left teeth 2–6 (left) and the histologic features of the lesion (right). Magnification: ×40. **b** GNAS mutation detection revealed no mutation of the codons Arg201 and Gln227. **c** Super-CNAs on Chr 12 were identified in LCM samples. **d** Amplification of MDM2 and CDK4 was detected.

A 32-year-old female patient was referred to our hospital in 2013 complaining of postoperative regrowth of swelling on the right side of the face. In 2010, she underwent her first maxillofacial surgery (trimming) in an external hospital, and the postoperative pathological diagnosis was FD of the jaw. Clinical examination revealed a hard and immobile mass with obvious tenderness on the right infraorbital and paranasal regions that was ill demarcated. Intraoral clinical examination showed a mass located on the right maxilla with buccal expansion involving upper left teeth 2 to 6. The skin covering the mass was intact. Her medical and family history was normal, and laboratory examination revealed no abnormality. Panoramic radiograph and spiral computed tomography (CT) (Fig. 5a) showed loss of the normal trabecular structure of the alveolar process corresponding to the right maxillary tuberosity and the maxilla with ‘ground-glass’ opacity, which resembled FD. Based on her disease history, a second trimming surgery was performed in our hospital in 2013. Histopathological examination of the trimmed specimen from this surgery revealed a lesion containing collagen fibers and irregular bone trabeculae and bone spicules, around which osteoblasts were observed, which was consistent with the features of OF. Considering the FD feature radiologically and OF feature pathologically, the patient was then diagnosed with a descriptive report as “a fibro-osseous lesion tending to be ossifying fibroma based on clinical manifestations, pathological and imaging features”.

Four years later, this patient came to our hospital again, complaining of another regrowth swelling on the right side of the maxilla after the last surgery. The treatment choice between trimming (best for FD) and complete resection (best for OF) was carefully discussed, and the latter was ultimately conducted, as mainly based on the patient’s previous treatment history and a pathological predilection diagnosis of OF. The patient has been followed up for three years, with a favorable prognosis.

In this case, it was obvious that an accurate diagnosis was critical for treatment and prognosis. Unfortunately, without further evidence, the diagnosis was challenging. We first attempted to improve her diagnosis by GNAS mutation detection, which revealed a wildtype status in both mutation hotspots reported for FD, which was inconclusive (Fig. 5b). We then applied morphologically assisted CNA analysis using LCM samples and discovered a supergain event (CN = 8.8–10, mean = 9.4) on Chr 12, consistent with previous observations (Fig. 5c). We also performed qPCR on extracted gDNA and detected significantly high amplification of MDM2 and CDK4 (10-fold for MDM2; 9-fold for CDK4) (Fig. 5d). Both results provide strong support for the diagnosis of OF, instead of the previously identified FD, of the jaw.

### Case study two: the potential role of CNA in the prediction of osteosarcoma

**Fig. 6:**
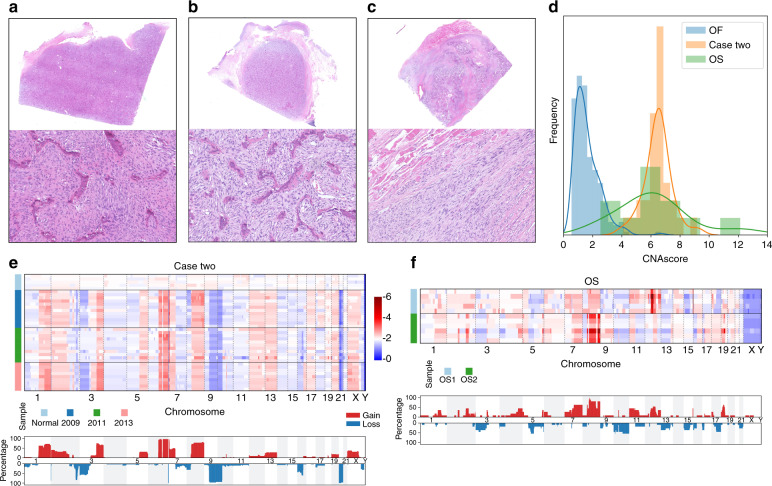
The association between CNA and malignant transformation in Case 2. **a**–**c** H&E staining images showing the tissue morphology of Case 2 in 2009 (**a**), 2011 (**b**), and 2013 (**c**). Magnification: ×12.5 (upper panels) and ×200 (lower panels). **d** Normalized frequency distribution of CNAscore in Case 2 and in OS and OF samples. **e** CNA profiles of 37 samples in Case 2. Heatmap (upper panel) and aggregation of copy number alterations (lower panel) show the key CNA events for specific chromosomes. For the lower panel, the *y* axis represents the percentage of samples harboring CNAs. **f** CNA profiles of 11 samples from two osteosarcoma patients. Heatmap (upper panel) and aggregation of copy number alterations (lower panel) show the key CNA events for specific chromosomes. For the lower panel, the *y* axis represents the percentage of samples harboring CNAs.

The frequent CNA hotspots in OF patients may be associated with the development of OF, and many reports have demonstrated the underlying roles of CNAs in cancer progression. We further studied a patient who was treated in our hospital three times: in 2009, 2011, and 2013. The patient was diagnosed pathologically with descriptive reports as a cellular fibro-osseous lesion in suspicion of OF for the first two times, and then the recurrent lesion in 2013 showed clear pathological signs of malignancy, and thus was diagnosed as osteosarcoma (OS) (Fig. 6a–c). No signs of genetic CNAs were observed when sequencing normal tissue samples from the patient. However, we detected severe copy number changes using fibrous and calcified tissues in 2009, 2011, and 2013 (Fig. 6e). Unlike the CNA patterns of OF samples, in which most copy number changes are present on only one chromosome, we found that copy number changes in this patient were widely distributed across the entire genome. We also detected breakpoints on Chr 1, Chr 2, Chr 3, Chr 5, Chr 6, Chr 7, Chr 8, Chr 9, and Chr 10, which were not observed in OF samples. The CNA patterns among the four years were consistent, with slight differences; this suggests that genome reorganization occurred long before pathological changes.

To further validate our findings, we collected 11 samples from two OS patients (Fig. 6f). Based on CNAscore, samples of the patient (case two) showed genome complexity similar to that in OS samples and significantly higher complexity than in OF samples (Fig. 6d). At the same time, similar breakpoint patterns on Chr 1, Chr 3, and Chr 8 were detected in OS samples.

## Discussion

OF and FD are diseases that often require differential diagnosis because of overlaps and similarities in their clinical, radiological, and morphological characteristics.^28^ This poses a dilemma for pathologists, especially in the absence of typical features and the frequent existence of histological alterations.^29^ Although previous research by our research group showed that the GNAS gene is a reliable adjunct for differentiating between OF and FD of the jaw,^30^ GNAS mutations can only be detected in 45%–88% of FD cases, leaving a challenging situation for undetected lesions. In addition, HPRT2 gene alteration in OF may not be a potential marker for diagnosis because of the low mutation rates.^5,31^ Further study at the molecular level is required to address this limitation. In this study, we applied LCM to preserve the spatial information of tissue sections and ensure the cellular purity and composition of cells in the sample. The advantages of this multiregional microdissected sequencing (MMS) technique, which combines LCM and next-generation sequencing, include a more accurate sampling of lesion tissues, and more sensitive detection of CNAs in minibulk tissue samples of ~30–50 cells. In addition, MMS also provides a useful tool to study genetic heterogeneity in different morphological areas of FD and OF lesions.

In general, genome copy number gains and losses in FD and OF of the jaw have rarely been studied. Analyses of chromosome aberrations in fibro-osseous lesions have failed to achieve satisfactory results due to the upgrading of experimental technology, the choice of experimental materials, and the scarcity of cases.^32,33^ Our results indicate that CNAs are frequent in OF of the jaw, with a detection rate of 44.8% in this study. Gains and losses occur frequently on Chrs 1, 3, 4, 5, 7, 9, 12, and 22. Our study also indicated that distinct genomic patterns of CNAs related to OF and FD, specifically the copy number amplifications on Chr 7 and Chr 12, are the most characteristic features of OF and together account for 44.8% of all CNA events. In comparison, there was no evidence for emerging copy number amplifications in 28 FD patients. Our results demonstrate that sequencing-based CNA analysis through the MMS approach is a novel and effective approach to differentiate OF and FD lesions. Of the signature CNAs on Chr 7 and Chr 12 in the OF cases, segmental amplifications and deletions were major features (69%) compared to small-size copy number changes.

We identified 8 CNA-associated genes that exhibit copy number amplifications in the smallest common CNA regions on Chr 7 and Chr 12. This finding agrees with a previous report of rearrangements on the long arm of Chr 12 in craniofacial OF according to qPCR.^28^ Recurrent molecular abnormalities in MDM2, located in the CNA region of Chr 12, have also been reported in juvenile OF.^28^ It is interesting to note that only one (P6) out of 4 patients, who had CNA on Chr 12, showed a significant difference in MDM2 and CDK4 amplifications (Fig. 3d). Both genes are located on the long arm (12q15 and 12q14.1 respectively) of Chr 12 reflecting the CNA abnormalities in P6 on both the short and long arms of Chr 12. The other 3 patients (P7, P8, and P9) only had CNA on the short arm of Chr 12 (Fig. 3b). Such a correlation between the CNA profile and individual patient suggests that MMS has great diagnostic potential when craniomaxillofacial fibro-osseous lesions lack typical morphological features. Of the two case reports, we used MMS to identify the copy number gain on Chr 12 in case one and then determined OF as a diagnosis. Since low-grade osteosarcoma can sometimes be confused with benign tumors such as OF and FD due to morphological overlaps,^34,35^ in case two, malignant transformation to OS occurred in a suspicious OF patient, and the identical CNA features across the samples collected at different time points suggest that genome CNAs can occur prior to phenotypic changes, revealing the potential of CNA analysis in the prediction of OS.

Of note, sixteen OF patients in our study carried no detectable CNAs, indicating that the diagnosis of OF cannot be ruled out when somatic CNA is absent. To date, the details of OF pathogenesis are not completely clear, and the causality between the occurrence of CNAs and tumor formation needs to be further studied. Currently, the mainstream model for copy number evolution assumes that CNAs are acquired gradually and sequentially for a long period of time,^36,37^ leading to successively more serious consequences. In addition, the somatic nature of CNAs in OFs may not satisfy this level of sensitivity when genomic alterations occur in a few cells. Therefore, we speculate that the tumor samples that did not show CNAs may have not undergone genomic changes by the time when the tumors were removed or they were insensitive to the detection technique. In any case, the fact that only 44.8% of OF cases in the present study showed detectable CNAs may limit this technology for an “everyday” test to differential diagnosis between OF and FD. We think that this technology is more useful for diagnostically challenging cases with ambiguous clinical and pathological features.

To the best of our knowledge, this is the first study to provide evidence of the significant difference between OF and FD based on genome-wide DNA copy number analysis. Molecular diagnosis overcomes the limitation of traditional pathological diagnosis. In our research, gDNA was extracted from single microconnected tissue and microcalcified tissue from patients with OF or FD (including McCune–Albright syndrome) and not from peripheral blood or bulk tissues. It is generally known that the extraction of calcified tissue DNA poses severe challenges for researchers because of the major pitfall of the quality and quantity of nucleic acids.^38–40^ Hence, the MMS approach should be taken into consideration for routine diagnostic use, as only a small amount of viable DNA is needed to provide reliable and satisfactory sequencing results for high-resolution CNA analysis. Although promising, due to the rarity of these two fibro-osseous lesions, the results of this study are based on CNAs in frozen tissues from 29 OF and 28 FD cases. Although the cost of the MMS approach is higher than that of Sanger sequencing, the former can provide more accurate diagnosis and better guide treatment plans. Next, we will conduct a similar study on a larger cohort and attempt to apply copy number analysis to paraffin-embedded tissues of fibro-osseous lesions to further confirm the specificity of CNA patterns in these diseases.

In conclusion, a multiregional microdissection sequencing approach will open a new avenue for differential diagnosis between fibrous dysplasia and ossifying fibroma of the jaw. Our study verifies the practicability and feasibility of using laser microdissection to capture a small number of morphologically well-defined cells for the identification of CNAs through high-throughput sequencing in the diagnosis of fibro-osseous lesions. Of particular interest are the findings of CNAs in 44.8% of OF patients; few CNAs were detected in FD patients. The distinct somatic CNA patterns associated with OF and FD of the jaw, particularly the copy number changes on Chr 7 and Chr 12, were the most distinctive and suggestive of OF. By performing copy number and qPCR analyses, eight novel genes were found to have differential copy number changes in OF and FD of the jaw. Although the diagnosis of OF could not be ruled out when somatic CNA was absent, copy number analysis via multiregional microdissection sequencing still holds great promise for accurately differentiating OF from FD of the jaw.

## Materials and methods

### Case selection and review

A total of 29 cases (220 samples) of OF and 28 cases (174 samples) of FD arising from the jaws, as well as their adjacent normal tissues, collected from 2008 to 2018 were obtained from the tissue bank of Peking University Hospital of Stomatology. Interestingly, malignant changes three years after surgery occurred in one case initially diagnosed as a cellular fibro-osseous lesion in suspicion of OF, and we included this case in our study. Two cases of primary osteosarcoma of the jaw were selected as controls. In addition, a case diagnosed as “tending to be ossifying fibroma” with overlapping pathologic features of FD and OF was also included. All samples were obtained with the approval of the Ethics Committee of the Peking University Hospital of Stomatology. Records were reviewed, and the diagnosis was confirmed by three experienced pathologists. Once removed, all fresh tissues were immediately stored in liquid nitrogen or at −80 °C. Detailed patient information is listed in Supplementary Table 1 and Tables 2, 3.

### Frozen tissue section staining and multiregional microdissection sequencing

1. Tissue section staining
  Frozen samples were embedded in O.C.T. compound. Mounted frozen samples were cut using a Leica Cryostat, and 10-μm-thick sections were placed on DNA/RNA-free enzyme-treated PEN membrane slides to prevent degradation of nucleic acids. The O.C.T. compound was then removed, and the tissue sections were stained with hematoxylin and eosin (H&E). Before cell capture, the sections were scanned by a scanner (Nano Zoomer Digital pathology scanner) at ×10 magnification. We reviewed adjacent 5-μm H&E-stained serial sections to obtain spatial information prior to LCM.
2. Minibulk sample isolation by laser catapulting
  To adjust the parameters of the Leica laser microcutting system to achieve optimum working conditions, the suitable energy for laser-catapulting minibulk samples was set as 100–120 delta. The connective tissue and calcified tissue containing 30–50 cells were cut directly into the caps of 200-μL PCR tubes, each containing 10 μL of DNA microextraction reagent by UV laser. For each tissue section, ten regions were selected as biological replicates. The captured tissues were lysed in 10 μL lysis buffer (8.535 μL nuclease-free water, 0.3 μL 1 mol·L^−1^ Tris-HCl, 0.025 μL 4 mol·L^−1^ NaCl, 0.1 μL 500 mol·L^−1^ EDTA, 0.04 μL Triton X-100 and 1 μL proteinase K (Qiagen 19133)) at 50°C for 14 h.
3. Library construction and sequencing

The DNA in the lysis buffer was tagmented by the Tn5 transposase at 55 °C for 2 h in a reaction containing 0.1 μL TTE Mix V50 (Vazyme, TD501), 0.25 μL 50× Proteinase Inhibitor (Promega, G6521), 4 μL 5× TD buffer, 0.2 μL 0.1 mol ·L^−1^ MgCl_2_, 5.45 μL Nuclease-free water, 10 μL lysis template. The sample was then amplified via 25 cycles of PCR (reaction mixture: 22 μL Q5 High-Fidelity 2× Master Mix (New England Biolabs, M0492), 1 μL 10 mmol·L^−1^ Nextera i5 primer, 1 μL 10 mmol· L^−1^ Nextera i7 primer, 10 μL tagmentation template) and purified using VAHTS DNA clean beads (Vazyme, N411). Typically, 2 µg of final amplified product was obtained for each sample. The concentration of each sample was measured using a Qubit system (Invitrogen), and the fragment size (typical size varies from 300 to 700 bp) was determined with a fragment analyzer (Agilent). All libraries were then sequenced using an Illumina HiSeq 4000 sequencer with PE150.

### DNA extraction from bulk tissues and qPCR

According to the results of OF genomic copy number analysis, the smallest common regions of recurrent CNAs in all samples were analyzed as described (Supplementary Tables 4 and 5).^41,42^ We selected possible target genes in the smallest common regions of the hotspot CNAs to examine CNA-associated gene amplification by qPCR, as described previously. qPCR amplification for CDK4 and MDM2 was carried out for four patients with OF and special case 1 (OF). qPCR amplification of HILPDA, CALD1, C1GALT1, MICALL2, PHF14, and AIMP2 was performed for three patients with OF as described.^43^ We used the albumin gene (ALB) as a reference. A standard curve was constructed based on DNA amplification of normal tissues, and the amplification fold of all target genes was calculated. The relative amounts of target genes were determined as a ratio of the albumin gene (ALB) and calculated with Light Cycler Relative Quantification software. The primer sequences used are listed in Supplementary Table 6.

### GNAS gene mutational analysis

All frozen tissues from 28 cases of FD and one case with a confusing diagnosis were used for GNAS mutation detection. DNA in tissues was extracted by a QIAamp DNA Mini Kit (Qiagen, Valencia, CA, USA) according to the manufacturer’s instructions. For each sample, 200 ng of genomic DNA was amplified using GoTaq Green Master Mix (Promega, Madison, WI, USA). PCR amplification and direct DNA sequencing were performed as described.^30^

### Sequencing data analysis

Adapter trimming was first performed on 2×150 paired-end reads by Cutadapt (version 2.10)^44^ and then aligned to the human reference genome (hg19) using Bowtie2 aligner (version 2.2.9).^45^ Approximately 1 mol·L^−1^ mapped reads were obtained for each sample. The reads were tabulated into nonoverlapping dynamic bins (1 Mb resolution) across the genome. Lowess regression normalization was performed to reduce GC bias in the bin counts. The copy number was called using the R package DNAcopy^46^ using the circular binary segmentation (CBS)^47^ algorithm (alpha = 0.1, min. width = 5, undo. SD = 0.1).

Median absolute pairwise differences (MAPD)^48^ were calculated to identify and filter out low-quality samples (MAPD ≥ 0.23). If *xi* is the copy number value of the ith bin, then

$${\mathrm{MAPD}} = {\mathrm{median}}(|x_{i + 1} - x_i|),$$

where *I* is ordered by the genomic position.

If *s*_*i*_ is the copy number value of the ith segment, then CNAscore is calculated by

$${\mathrm{CNA}}\,{\mathrm{score}} = \mathop {\sum }\limits_{{\mathrm{chr}} = 1}^{24} ({\mathrm{mean}}(\left| {s_i - s_{i - 1}} \right|) + 0.5 \ast |{\mathrm{mean}}\left( {s_i} \right) - {\mathrm{norm}}|)$$

where *i* is ordered by the genomic position and norm is the neutral copy number of each segment (norm = 0, 1 or 2).

For diploid chromosomes, copy number gain was defined as CN > 2.4 and copy number loss as CN < 1.7. For haploid chromosomes, copy number gain was defined as CN > 1.5 and loss as CN < 0.5.

### Statistical analysis

All statistical analyses were performed using either SPSS 23.0, R v3.3.1 (The R Foundation for Statistical Computing) or Python v3.8.3. *P* ≤ 0.05 was considered statistically significant. Appropriate descriptive statistics were used in the study.

## Supplementary information

Supplementary information
