# Supplementary material for: Copy number alteration profiling facilitates differential diagnosis between ossifying fibroma and fibrous dysplasia of the jaws
Source: Int J Oral Sci. 2021 Jun 30;13:21. doi: 10.1038/s41368-021-00127-3 (PMC8242074; doi:10.1038/s41368-021-00127-3)
Supplement: Supplementary file 1 — Supplementary information [file 41368_2021_127_MOESM1_ESM.pdf]

Table 1 Clinical and pathological findings and CNA patterns in patients with OF

No.	onset age (years)	Operation age (years)	Duration (years)	sex	site	CNA(chromosome)	No.of samples(CNA/total)
1	23	24	1	F	maxilla	Chr7	9(9)
2	8	8	0.1	M	maxilla	Chr7	9(10)
3	12	14	1.5	M	mandible	Chr7	7(7)
4	8	8	0.75	M	mandible	Chr7	8(8)
5	32	32	0.1	F	maxilla	Chr7	6(6)
6	20	22	2	M	mandible	Chr5/12	7(8)
7	8	8	0.25	M	mandible	Chr12	6(8)
8	21	22	1	F	mandible	Chr12	2(3)
9	39	39	0.1	F	mandible	Chr12	8(8)
10	11	11	0.1	F	maxilla	Chr22	8(8)
11	18	25	7	M	mandible	Chr4	9(9)
12	14	14	0.1	M	mandible	Chr3/6/9/13/21	7(7)
13	52	53	1	F	mandible	Chr1	10(12)
14	38	40	2	M	maxilla	CNA-free	0(11)
15	50	53	3	F	mandible	CNA-free	0(4)
16	9	9	0.5	M	maxilla	CNA-free	0(8)
17	42	44	2	M	mandible	CNA-free	0(10)
18	5	5	0.1	M	maxilla	CNA-free	0(9)
19	20	20	0.1	F	mandible	CNA-free	0(9)
20	22	22	0.25	F	Maxilla	CNA-free	0(8)
21	45	47	2	M	mandible	CNA-free	0(4)
22	25	27	2	F	mandible	CNA-free	0(7)
23	32	32	0.67	F	mandible	CNA-free	0(8)
24	12	12	0.25	F	mandible	CNA-free	0(8)
25	31	31	0.1	M	mandible	CNA-free	0(6)
26	23	23	0.2	F	mandible	CNA-free	0(10)
27	61	62	0.5	F	mandible	CNA-free	0(6)
28	10	17	7	F	mandible	CNA-free	0(6)
29	10	16	6	M	maxilla	CNA-free	0(3)

Abbreviations: F , female; M, male.

Table 2 Clinical and pathological findings and CNApatterns in patients with FD

No.	onset age (years)	Operation age(years)	Duration (years)	sex	site	CNA(Chromosome)	No.of samples(CNA/total)
1	8	18	10	M	both	CNA-free	0(5)
2	8	18	10	M	maxilla	CNA-free	0(8)
3	2	6	4	F	both	CNA-free	0(7)
4	9	12	3	F	both	CNA-free	0(5)
5	28	32	4	F	both	CNA-free	0(6)
6	17	27	10	M	maxilla	CNA-free	0(2)
7	17	47	30	M	both	CNA-free	0(6)
8	22	37	15	M	mandible	CNA-free	0(1)
9	8	18	10	F	maxilla	CNA-free	0(8)
10	43	48	5	M	mandible	CNA-free	0(9)
11	8	27	19	F	both	Chr7(loss)	6(6)
12	15	18	3	M	mandible	CNA-free	0(3)
13	14	21	7	M	maxilla，zygoma	CNA-free	0(6)
14	7	23	16	M	maxilla	CNA-free	0(7)
15	38	38	0.1	F	maxilla	CNA-free	0(6)
16	4	24	20	M	maxilla	CNA-free	0(1)
17	11	17	6	M	maxilla	CNA-free	0(5)
18	40	40	0.5	F	mandible	CNA-free	0(5)
19	13	28	15	F	maxilla	CNA-free	0(6)
20	38	38	0.1	F	maxilla	CNA-free	0(7)
21	22	22	0.2	M	mandible	CNA-free	0(9)
22	5	21	16	F	both	CNA-free	0(8)
23	17	19	2	M	maxilla，zygoma	CNA-free	0(9)
24	16	21	5	M	maxilla	CNA-free	0(8)
25	13	28	15	F	maxilla	CNA-free	0(7)
26	20	24	4	M	maxilla	CNA-free	0(8)
27	12	25	13	F	Maxilla	CNA-free	0(8)
28	15	15	0.5	M	Maxilla	CNA-free	0(8)

Abbreviations: F , female; M, male.
‘Both’ indicates that the lesion occurred in both mandible and maxilla.

Table 3 Clinical and pathological findings and CNA patterns in patients with two special cases and two OS cases

No.	onset age (years)	Operation age (years)	duration(years)	sex	site	CNA(Chromosome)
case1	32	32/36	0.5/4	F	maxilla	Chr12
case2	41	41/43/45	0.1/1.3/2	F	maxilla	all chromosomes
OS1	73	73	0.5	M	mandibular	all chromosomes
OS2	68	68	0.25	M	maxilla	all chromosomes

Table 4 Smallest common regions of recurrent copy number gain

Aberration	Position(strat)	Position(end)	Possible target genes
+7p22.3	10157	2800000	FAM20C,c7orf50,MICALL2,ZFAND2A,EIF3B,LFNG,GNA12,BRAT1,CHST12,IQCE,TTYH3,PDGFA,PSMG3,ADAP1,MRM2
+7p22.1	5110329	6466458	AIMP2,KDELR2,WIP12,SLC29A4,TNRC18,FBXL18,RNF216,PMS2,USP42,FAM220A,CCZ1,CYTH3,FSCN1
+7p22.1-p14.3	7182615	32243547	C1GALT1,PHF14,ICA1,ETV1,CRPPA,ITGB8,PDE1C,SCIN,FAM126A,TAX1BP1,GARS,AQP1,IGF2BP3,PLEKHA8,CRHR2
+7q31.32-q34	122890421	139645852	TNPO3,PLXNA4,AHCYL2,GRM8,POT1,HILPDA,CALD1,SND1,EXOC4,PTN,AKR1B1,IQUB
+12p13.33-p13.32	1923610	3784481	CACNA1C,FKBP4,FOXM1,PRMT8
+12p13.32	4073204	4525437	CCND2,TIGAR,FGF23,C12orf4
+12p13.32-p13.31	4753800	5338045	GALNT1,KCNA1,KCNA5
+12p13.31	5561279	5754449	ANO2
+12p12.1-p11.23	25000000	26607141	KRAS
+12p11.22	28585006	29184822	FAR2
+12q13.2-q14.2	55565556	63123955	CDK4,METTL1,GLI1,ITGA7,R3HDM2
+12q14.2-q21.1	64509370	72765143	MDM2,LEMD3,CAND1,RAP1B,CPM,FRS2,CNOT2,RAB3IP,HMGA2,YEATS4

+, copy number amplification. −, copy number deletion

Table 5 Smallest common regions of recurrent copy number loss

Aberration	Position(strat)	Position(end)	Possible target genes
-1q31.1-q31.2	190732700	193046426	RSG1,RSG2,RSG18,UCHL5
-4q12-q21.22	52790298	82529829	LNX1,EPHA5,SLC4A4,ANXA3,BMP2K,BMP3,HNRNPD,PRKG2,YTHDC1
-7p14.3-p11.2	33680849	54408146	AMPH,STK17A
-7p11.2-q11.23	55592885	77806042	-
-7q21.11-q22.3	78959487	1066278876	SEMA3D,LRRC17
-12p12.1-p11.23	26022384	26430092	RASSF8,BHLHE41,SSPN,ITPR2
-12p11.22	29431322	29805715	TMC1,OVCH1
-12p11.21-p11.1	31084637	33432935	DDX1,DNM1L
-12q12	44157936	44514689	TMEM117
-12q12-q13.12	44979147	49397803	DBX2,RPAP3,HDAC7,PFKM,CCNT1,PRKAG1
-12q13.13-q13.2	54273593	54802232	CBX5,COPZ1,NFE2,ITGA5,PDE1B,DCD
-22q11.1-q13.33	17274108	51019200	Chromosome 22 long arm rearrangement

+, copy number amplification. −, copy number deletion

Table 6 Sequences of primers used for qpcr reaction

Gene (locus)	Sequence	Function
MDM2-F(12q15)	5'-CCGGAT GATCGCAGGTG-3'	proto-oncogene
MDM2-R	5'-AAAAGCTG AGTCAACCTGCCC-3'	
CDK4-F(12q14.1)	5'-ATGGCTACCTCTCGATATGAGC-3'	cancer and sarcoma
CDK4-R	5'-CATTGGGGACTCTCACACTCT-3'	
HILPDA-F(7q32.1)	5'-AAGCATGTGTTGAACCTCTACC-3'	epithelial ovarian cancer,clear cell adenocarcinomas
HILPDA-R	5'-TGTGTTGGCTAGTTGGCTTCT-3'	
CALD1-F(7q33)	5'-TCGACCCAACAATAACAGATGC-3'	primary non-muscle-invasive bladder cancer,colon adenocarcinoma and lymph node metastases
CALD1-R	5'-TCTCGTATCTTTCTTGGCGACT-3'	
C1GALT1-F(7p22.1-p21.3)	5'-TCCTCTGTGGATCAGCAATAGG-3'	head and neck cancer
C1GALT1-R	5'-TTAGGCTGGGTGTCAACCTTT-3'	
MICALL2-F(7p22.3)	5'-GCCCTCGTGTGACCAATAGC-3'	gastric cancer
MICALL2-R	5'-GTGGAGCTTGAACTGAGTGTG-3'	
PHF14-F(7p21.3)	5'-AGGGAAGCGGGAGTGATGAA-3'	lung cancer
PHF14-R	5'-GGTCAGGCTATCATTGGTCAGT-3'	
AIMP2-F(7p22.1)	5'-TGCAAGCTCTTGAGTCCCG-3'	nasopharyngeal carcinoma
AIMP2-R	5'-ACATCCAAGTCTGCATCTGGT-3'	
